# Epidemiology of healthcare-associated bloodstream infection in South African neonatal units

**DOI:** 10.1186/s12879-024-10219-0

**Published:** 2024-11-26

**Authors:** Angela Dramowski, Larisse Bolton, Adrie Bekker, Arnoldus Engelbrecht, Louisa Erasmus, Aaqilah Fataar, Chandre Geldenhuys, Marlize Kunneke, Dave Le Roux, Natasha O’ Connell, Kessendri Reddy, Natasha Rhoda, Lloyd Tooke, Mark Wates, Thandi Wessels, Cari van Schalkwyk, Andrew Whitelaw

**Affiliations:** 1 Department of Paediatrics and Child Health, Faculty of Medicine and Health Sciences, Stellenbosch University, PO Box 241, Cape Town, 8000 South Africa; 2 South African Centre for Epidemiological Modelling and Analysis (SACEMA), School of Data Science and Computational Thinking, Stellenbosch University, Stellenbosch, South Africa; 3 Division of Medical Microbiology and Immunology, Department of Pathology, Faculty of Medicine and Health Sciences, Stellenbosch University, Cape Town, South Africa; 4 Department of Paediatrics, Worcester Provincial Hospital, Worcester, South Africa; 5 Department of Paediatrics, Paarl Hospital, Paarl, South Africa; 6 Department of Paediatrics, New Somerset Hospital, Cape Town, South Africa; 7 Department of Paediatrics, Khayelitsha District Hospital, Cape Town, South Africa; 8 Department of Neonatology, Mowbray Maternity Hospital, Cape Town, South Africa; 9 School of Child and Adolescent Health, Faculty of Health Sciences, University of Cape Town, Cape Town, South Africa; 10 Department of Neonatology, Groote Schuur Hospital, Cape Town, South Africa; 11 Department of Paediatrics, Karl Bremer Hospital, Cape Town, South Africa

**Keywords:** Neonate, Sepsis, Bloodstream infection, Healthcare-associated infection, Hospital-acquired infection, Nosocomial, Antimicrobial resistance, Neonatal intensive care unit

## Abstract

**Background:**

Reports of healthcare-associated bloodstream infection (HA-BSI) epidemiology in African neonatal units are limited.

**Methods:**

We conducted a cross-sectional study (2017–2018) in nine neonatal units in the Western Cape Province, South Africa, including central, regional and district hospitals (416 beds) using laboratory and clinical records. Patient demographics, HA-BSI rates, pathogen spectrum, and hospital outcomes and empiric antibiotic coverage rates were determined.

**Results:**

Over two years, 23,748 neonates were admitted with unit occupancy rates ranging from 79 to 93%. 485 HA-BSI episodes occurred, with median onset at 11 (IQR 7–24) days of life. Most HA-BSI episodes (348; 72%) affected very low birth weight neonates (< 1500 g). The overall HA-BSI rate was 2.0/1000 patient days. The highest HA-BSI rate was observed at the central unit with onsite surgery (3.8/1000 patient days). Crude HA-BSI mortality was 31.8% (154/485) with two-thirds of deaths occurring within three days of BSI onset. Higher mortality was observed for Gram-negative/fungal BSI compared to Gram-positive BSI (RR 1.5; 95%CI 1.1-2.0; *p* = 0.01) and very preterm neonates (gestation < 32 weeks) versus ≥ 32 weeks (RR 1.5; 95%CI 1.1–2.1; *p* = 0.01). Mean estimated empiric antibiotic coverage rates varied by unit type: 66–79% for piperacillin-tazobactam plus amikacin, 60–76% for meropenem and 84–92% for meropenem plus vancomycin.

**Conclusion:**

Most HA-BSI events affected preterm neonates at the central hospital with onsite surgery. One-third of patients with HA-BSI died, with highest mortality in preterm infants and Gram-negative/fungal BSI. Empiric antibiotic regimens provide moderate coverage of circulating pathogens but require annual review given increasing carbapenem resistance rates.

**Supplementary Information:**

The online version contains supplementary material available at 10.1186/s12879-024-10219-0.

## Background

Worldwide, an estimated 2.3 million newborns die in their first 28 days of life each year [1]. There is gross disparity in the regional distribution of neonatal deaths with 80% occurring in Sub-Saharan Africa and South Asia [2, 3]. The leading causes of neonatal death remain prematurity-related complications (35%), infection-related events (27%) and intrapartum-related events (23%) [1].

Healthcare-associated bloodstream infections (HA-BSI, occurring on/after day 3 of life), are a major contributor to infection-related neonatal deaths, especially in small and sick hospitalised newborns [4, 5]. Risk factors for developing neonatal HA-BSI include preterm birth, low birth weight, surgical comorbidity, prolonged hospital stay, antibiotic exposure, skin and gastrointestinal barrier disruption, and use of indwelling devices [6]. Healthcare systems and infrastructural challenges such as overcrowding, lack of isolation rooms, lack of provision for hand hygiene, understaffing and sub-optimal infection prevention and control (IPC) practices are additional factors driving HA-BSI rates in low- and middle-income countries (LMIC) [7, 8].

Reports of HA-BSI incidence in LMIC neonatal units range from 15 to 62 episodes per 1000 patient-days, which is nearly ten-fold that reported from high-income country units [9]. Globally, an estimated 3 million cases of neonatal sepsis (including HA-BSI and meningitis) occur annually with reported mortality rates ranging from 11 to 19% [10, 11]. The contribution of HA-BSI to overall neonatal mortality rates in LMIC may however be underestimated, as up to 70% of deaths ascribed to prematurity were linked to antimicrobial resistant (AMR) Gram-negative infections in a South African tertiary hospital neonatal unit [12]. Descriptions of HA-BSI epidemiology in South African neonatal units are limited, with incidence rates reported from tertiary hospitals in Cape Town (4 per 1000 patient days) [13, 14] and Johannesburg (15 per 1000 patient days) [15]. The first report of South African national neonatal incidence risk for laboratory-confirmed HA-BSI and/or meningitis estimated 4·9 episodes of per 1000 livebirths between 2014 and 2019 [16].

Emergence of AMR pathogens is a major concern for LMIC neonatal units where antimicrobial treatment options are limited. Regular evaluation of neonatal BSI pathogen spectrum and AMR patterns is essential as these change over time, and vary by region, practice setting and patient risk profile [17]. Given the paucity of data from African neonatal units, we described the epidemiology of HA-BSI in the Western Cape Province of South Africa to estimate HA-BSI rates, describe pathogen spectrum, hospital outcomes and empiric antibiotic coverage rates.

## Methods

### Study setting and design

The nine largest neonatal units in the Western Cape Province of South Africa, offering a total of 416 inpatient neonatal beds were included: three district units (Helderberg, Khayelitsha District, and Karl Bremer Hospitals), three regional units (New Somerset, Paarl, and Worcester hospitals), and three central units (Groote Schuur and Mowbray Maternity Hospitals both with off-site surgery, and Tygerberg Hospital with on-site surgery). Central hospitals provide specialised neonatal care, including neonatal intensive care with sub-specialist support; peripheral hospitals (regional and district) provide neonatal care by general paediatricians, with no or minimal neonatal intensive care and no neonatal surgery. Given the very limited neonatal intensive care beds available, most care of neonates requiring surfactant administration and non-invasive ventilation is provided in the general neonatal wards. A retrospective descriptive analysis of laboratory confirmed HA-BSI episodes (i.e. blood culture collection occurring on or after day 3 of neonatal unit admission) was undertaken for the period 1 January 2017 to 31 December 2018. All hospitalised patients (0-100 days) on any neonatal ward including neonatal intensive care (NICU), high care or acute care wards and kangaroo mother care wards with a culture-confirmed episode of HA-BSI were included, as extremely preterm neonates often require prolonged hospital stays. The Stellenbosch University Health Research Ethics Committee and Tygerberg Hospital management reviewed and approved the study (N18/07/068 and N20/07/070), with all hospitals providing approval for the study.

### Clinical management of HA-BSI episodes

Symptoms and signs that would prompt evaluation for suspected HA-BSI in these neonatal units include apnoea, temperature instability, respiratory distress, heart rate instability and feeding problems, among others. Infants with suspected HA-BSI are commenced empirically on piperacillin-tazobactam plus amikacin, or meropenem if clinically unstable or when meningitis is suspected, and meropenem plus vancomycin for patients at risk of methicillin-resistant *Staphylococcus aureus* infection e.g. recent use of a central line, or thrombophlebitis.

### Laboratory investigation of HA-BSI episodes

For infants with a suspected HA-BSI episode, clinicians submit a full blood count with differential count and a C-reactive protein test. At least one blood culture specimen is aseptically collected, inoculating a minimum of 1 mL of blood. Cerebrospinal fluid and urine for microscopy, culture and susceptibility testing is submitted to the laboratory at the attending clinician’s discretion. Blood cultures from the nine neonatal units are processed centrally at the National Health Laboratory Service (NHLS) microbiology laboratories located at Tygerberg and Groote Schuur Hospitals using automated BacT/Alert blood culture incubation systems and BacT/Alert PF Plus bottles (bioMerieux, Marcy l’E`toile, France). For positive blood cultures, a Gram stain is performed from the blood culture broth and the broth is sub-cultured and incubated overnight. Clinicians are telephonically alerted to potential pathogens observed on the Gram stain. Identification and susceptibility testing are performed using the automated VITEK 2 system (bioMerieux, Marcy l’E`toile, France) and/or disk diffusion testing, interpreted using the annually published Clinical Laboratory Standards Institute (CLSI) breakpoints [18]. Categorisation of likely resistance mechanisms is based on the data generated by the routine phenotypic susceptibility testing.

### Data sources and definitions

Diagnostic pathology records were obtained from the Central Data Warehouse of the NHLS for blood culture pathogen identification and antimicrobial susceptibility profiles. Organisms were classified as pathogens or probable contaminants using the United States Centre for Disease Control (US CDC) commensal list [19]. Coagulase negative staphylococci were deemed to be pathogens if two isolates of the same species were cultured from two separate specimens within two calendar days. Data was deduplicated by removing blood culture specimen results where the same pathogen was isolated within 14 days of the original specimen.

Clinical data (birthweight, gestational age at birth, day of life at HA-BSI onset, length of stay and hospital outcome) was provided by the lead clinician at each neonatal unit, using hospital records. Neonatal unit patient days and unit bed occupancy rates were obtained from the Provincial Health Data Centre. The following standard definitions were used to stratify the cohort: low birthweight (< 2500 g), very low birthweight (< 1500 g), extremely low birthweight (< 1000 g), extremely preterm (< 28 weeks), very preterm (28 - <32 weeks), moderate to late preterm (32–37 weeks) and term (38 weeks or more). BSI-attributable deaths were defined as death within three calendar days of the HA-BSI episode [20]. HA-BSI rates, blood culture contamination rates, pathogen spectrum, empiric antibiotic coverage estimates, and hospital outcomes were determined.

### Calculation of empiric antibiotic coverage rates

Empiric antibiotic coverage rates for three commonly used HA-BSI treatment regimens, piperacillin-tazobactam plus amikacin (PIP-AMIK), meropenem (MERO) alone, and meropenem plus vancomycin (MERO-VANC) were calculated with 95% credible intervals, using a weighted-incidence syndromic combination antibiogram (WISCA) [21] and stratified by hospital type. WISCAs are an advanced type of antibiogram targeting a single infection syndrome e.g. BSI, that calculates antibiotic coverage rates accounting for the relative frequency of different circulating pathogens and their antibiotic susceptibility profiles in a particular data sample. The method uses a Bayesian approach, assuming incomplete knowledge of the frequency of BSI causative bacteria and their antibiotic susceptibility profiles. Data variables included in the WISCA were total numbers of pathogens isolated from blood cultures (including all individual pathogens isolated from monomicrobial and polymicrobial blood cultures), the number of pathogens from each of the leading bacterial species and the number of isolates that underwent susceptibility testing to the antibiotic regimens of interest (PIP-AMIK, MERO, MERO-VANC) using the method described by Bielicki et al. (eAppendix) [22].

### Statistical analysis plan

Descriptive analyses were used to compare neonatal demographic characteristics with absolute number, percentage, median and interquartile range (IQR). Continuous data were tested for statistical significance using T-tests and categorical data, using Chi-square or Fisher’s exact test. A p-value of less than 0.05 was considered statistically significant. Analysis was stratified by neonatal unit type: central hospital with onsite surgery (*n* = 1), central hospital without onsite surgery (*n* = 2) and district or regional hospitals (*n* = 6). Empiric antibiotic coverage rates were reported as point estimates with 95% credible intervals calculated using Monte Carlo simulations, based on 1000 runs. The data was analysed in R Studio version 2022 running R statistical software version 4.1.0 [23]. The R packages employed during analysis was: tidyverse (1.3.2), lubridate (1.8.0), janitor (2.1.0), openxlsx (4.2.5), RVAidMemoire (0.9–81.2) and broom (1.0.1) [24].

## Results

### Characteristics of neonatal HA-BSI

Over two years, 23,748 neonates were admitted to the nine neonatal units with unit occupancy rates ranging from 79 to 93% (Table 1).

**Table 1:** Healthcare-associated bloodstream infection (HA-BSI) and blood culture sampling rates by neonatal unit type (2017–2018)

	Total sample	Central (medical + onsite surgery)	Central (medical + offsite surgery)	District + Regional (medical only)
**Neonatal unit profiles**	9 hospitals	1 hospital	2 hospitals	6 hospitals
Total beds	416	124	138	154
Mean neonatal unit occupancy rate	87.0%	92.7%	79.0%	90.4%
Total neonatal unit admissions	23,748	5672	7447	10,629
Total neonatal unit inpatient days	240,640	83,873	69,470	77,174
Mean hospital stay, days	10.1	14.8	9.3	7.3
**Blood culture sampling** ^**1**^				
Total blood cultures submitted	12,258	4047	3644	4567
Blood cultures per 100 admissions	51.6	71.4	48.9	43.0
Blood culture contamination rate^2^	240 (2.0%)	120 (3.0%)	9 (0.2%)	111 (2.4%)
**HA-BSI rates**				
Total HA-BSI episodes	485	315	84	86
HA-BSI incidence density^3^	2.0	3.8	1.2	1.1

HA-BSI = Healthcare-associated bloodstream infection

^1^ Blood culture sampling ≥ day 3 of life for suspected HA-BSI episodes

^2^ Blood culture contamination rate = number of cultures yielding contaminant/s per 100 blood cultures submitted

^3^ HA-BSI incidence density = (number of HA-BSI episodes/total neonatal inpatient days) x 1000

A total of 12258 blood cultures were collected for suspected HA-BSI episodes, with a sampling rate (from day 3 of life onwards) of 51.6 blood cultures per 100 neonatal unit admissions. Blood culture sampling rates were substantially higher at the central hospital with onsite surgery, compared to the other eight neonatal units (4047/5672 [71.4/100 admissions] vs. 8211/18076 [45.4/100 admissions; *p* < 0.001).

**Fig. 1:**
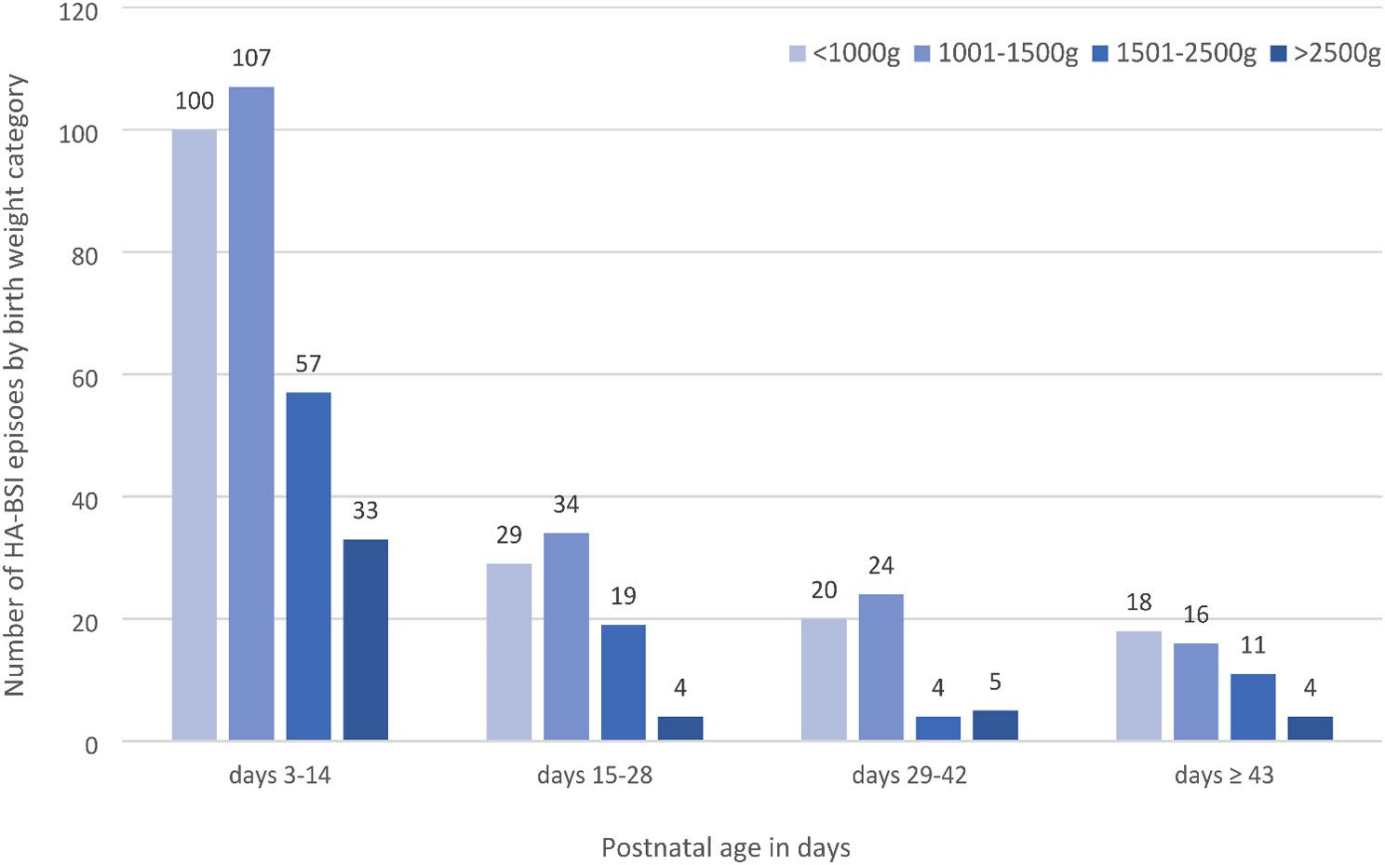
Healthcare-associated bloodstream infection (HA-BSI) episodes by birth weight category and postnatal age (*n* = 485)

In total, 485 HA-BSI episodes occurred with most (315; 64.9%) observed at the central neonatal unit with onsite surgery. HA-BSI onset occurred at a median of 11 days of life (IQR 7–24), with most cases arising in neonatal wards (73%) rather than the intensive/high care units (Table 2). Most HA-BSI episodes (338; 69.6%) affected extremely or very preterm infants. The median birthweight of infants with HA-BSI episodes was 1140 g (IQR 915–1550). Most HA-BSI episodes (297/485; 61.2%) were clustered in postnatal days 3–14 across all birth weight categories (Fig. 1).

**Table 2:** Profile of neonatal healthcare-associated bloodstream infection (HA-BSI) by unit type

	Total neonatal units (9 hospitals)	Central (onsite surgery) (1 hospital)	Central (offsite surgery) (2 hospitals)	District + Regional (medical only) (6 hospitals)	*p*-value
Total HA-BSI episodes	485	315	84	86	-
Pathogen load, n (%)					
monomicrobial polymicrobial	421 (86.3) 64 (13.2)	269 (85.4) 46 (14.6)	75 (89.3) 7 (8.3)	77 (89.5) 11(12.8)	0.318
Total HA-BSI pathogens, n (%) included in WISCA model not included in WISCA model	549 496 (90.3) 53 (9.7)	364 328 (90.1) 36 (9.9)	90 82 (91.1) 8 (8.9)	95 85 (89.5) 10 (10.5)	**-**
HA-BSI pathogen type, n (%)					
gram negative gram positive fungal	353 (64.3) 181 (33.0) 15 (2.7)	226 (62.1) 127 (34.9) 11 (3.0)	67 (74.4) 22 (24.4) 1 (1.2)	60 (63.2) 32 (33.7) 3 (3.1)	0.347
Median (IQR) HA-BSI onset, day	11 (7–24)	11 (7–25)	11 (6–23)	11 (7–18)	0.959
Location at HA-BSI onset					
neonatal ward intensive/high care unit	350 (72.2) 135 (27.8)	208 (66.0) 107 (34.0)	61 (72.6) 23 (27.4)	81 (94.2) 5 (5.8)	< 0.001
Gestational age, n (%)					
< 28 weeks 28–31 32–37 ≥ 38	125 (25.8) 213 (43.9) 103 (21.2) 44 (9.1)	72 (22.9) 133 (42.2) 80 (25.4) 30 (9.5)	36 (42.9) 37 (44.0) 7 (8.3) 4 (4.8)	17 (20.0) 43 (50.0) 16 (18.5) 10 (11.5)	< 0.001
Median (IQR) birth weight, grams	1140 (915–1550)	1165 (915–1670)	1055 (875–1263)	1163 (970–1480)	0.001
Median (IQR) hospital stay, days	35 (14–56)	37 (18–59)	31 (10–58)	27 (12–50)	0.01
Crude HA-BSI mortality, n (%)	156 (32.2%)	101 (32.1%)	29 (34.5%)	26 (30.2%)	0.83
HA-BSI-attributable mortality^1^	103/154 (66.9%)	63/100 (63.0%)	20/28 (71.4%)	20/26 (76.9%)	0.352
Mortality risk by gestational age at birth, n (%)					
< 28 weeks 28–31 32–37 ≥ 38	45/125 (36.0) 77/213 (36.1) 24/103 (23.3) 10/44 (22.6)	22/72 (30.6) 54/133 (47.8) 20/80 (25.0) 5/30 (16.7)	15/36 (41.7) 10/37 (27.0) 1/7 (14.3) 3/4 (75.0)	8/17 (47.1) 13/43 (30.2) 3/16 (18.8) 2/10 (20.0)	< 0.001
Mortality^2^ by HA-BSI type, n (%)					
gram negative gram positive fungal	113/307(36.8) 39/164 (23.8) 4/14 (28.6)	73/193 (37.8) 25/112 (22.3) 3/10 (30.0)	23/62 (37.1) 6/21 (28.6) 0/1 (0)	17/52 (32.7) 8/31 (25.8) 1/3 (33.3)	< 0.001
Relative frequency of the leading HA-BSI pathogens by unit type	*n* = 549	*n* = 364	*n* = 90	*n* = 952	-
*K. pneumoniae* *S. aureus* *E. coli* *S. marcescens* *A. baumannii* *E. faecalis* *E. faecium* *E. cloacae* *S. agalactiae* * P. aeruginosa* *Candida species*	140 (25.5) 87 (15.8) 56 (10.2) 52 (9.5) 42 (7.7) 36 (6.6) 34 (6.2) 25 (4.6) 22 (4.0) 18 (3.3) 15 (2.7)	85 (23.3) 70 (19.2) 26 (7.1) 39 (10.7) 31 (8.5) 24 (6.6) 23 (6.3) 20 (5.5) 10 (2.7) 12 (3.3) 11 (3.0)	27 (30.0) 6 (6.7) 17 (18.9) 6 (6.7) 9 (10.0) 6 (6.7) 4 (4.4) 2 (2.2) 6 (6.7) 4 (4.4) 1 (1.1)	8 (29.5) 11 (11.6) 13 (13.7) 7 (7.4) 2 (2.1) 6 (6.3) 7 (7.4) 3 (3.2) 6 (6.3) 2 (2.1) 3 (3.2)	

HA-BSI = healthcare-associated bloodstream infection; IQR = interquartile range; ^1^ HA-BSI attributable mortality = death within 3 days of HA-BSI onset; ^2^ Mortality by HA-BSI type: for calculation of mortality risk of polymicrobial infections, HA-BSI episodes with any Gram-negative pathogens and growth of either Gram-positive or fungi, were allocated to the Gram-negatives

Point estimate shown with 95% credible intervals, as denoted by error bars

### HA-BSI rates and profile by neonatal unit type

The overall HA-BSI rate was 2.0/1000 patient days, with the highest infection rate observed at the central unit with onsite surgery (3.8/1000 patient days compared to 1.2 and 1.1/1000 patient days at the central hospitals without onsite surgery and the district/regional hospitals respectively; *p* < 0.001). The overall blood culture contamination rate was 2% (240/12258).

From the 485 HA-BSI episodes, 549 individual pathogens were isolated as 65 episodes (13.2%) were polymicrobial BSI. Gram-negative pathogens predominated (353/549; 64.3%) followed by Gram positives (181/549; 33.0%) and fungi (15/549; 2.7%). *K. pneumoniae* (140, 28.2%), *S. aureus* (87; 17.5%), *E. coli* (56; 11.3%), *S. marcescens* (52; 10.5%) and *A. baumannii* (42; 8.5%) were the most prevalent HA-BSI pathogens. Crude HA-BSI mortality was 31.8% (154/485), with two-thirds of deaths (103/154; 66.9%) being BSI-attributable i.e. occurring within 3 days of BSI onset. Gram negative/fungal infections and gestational age < 32 weeks were associated with increased mortality risk compared to Gram-positive HA-BSI (RR 1.5; 95% CI 1.1-2.0; *p* = 0.01) and gestation ≥ 32 weeks (RR 1.5; 95% CI 1.1–2.1; *p* = 0.01).

### Empiric antibiotic coverage estimates by hospital type

**Fig. 2:**
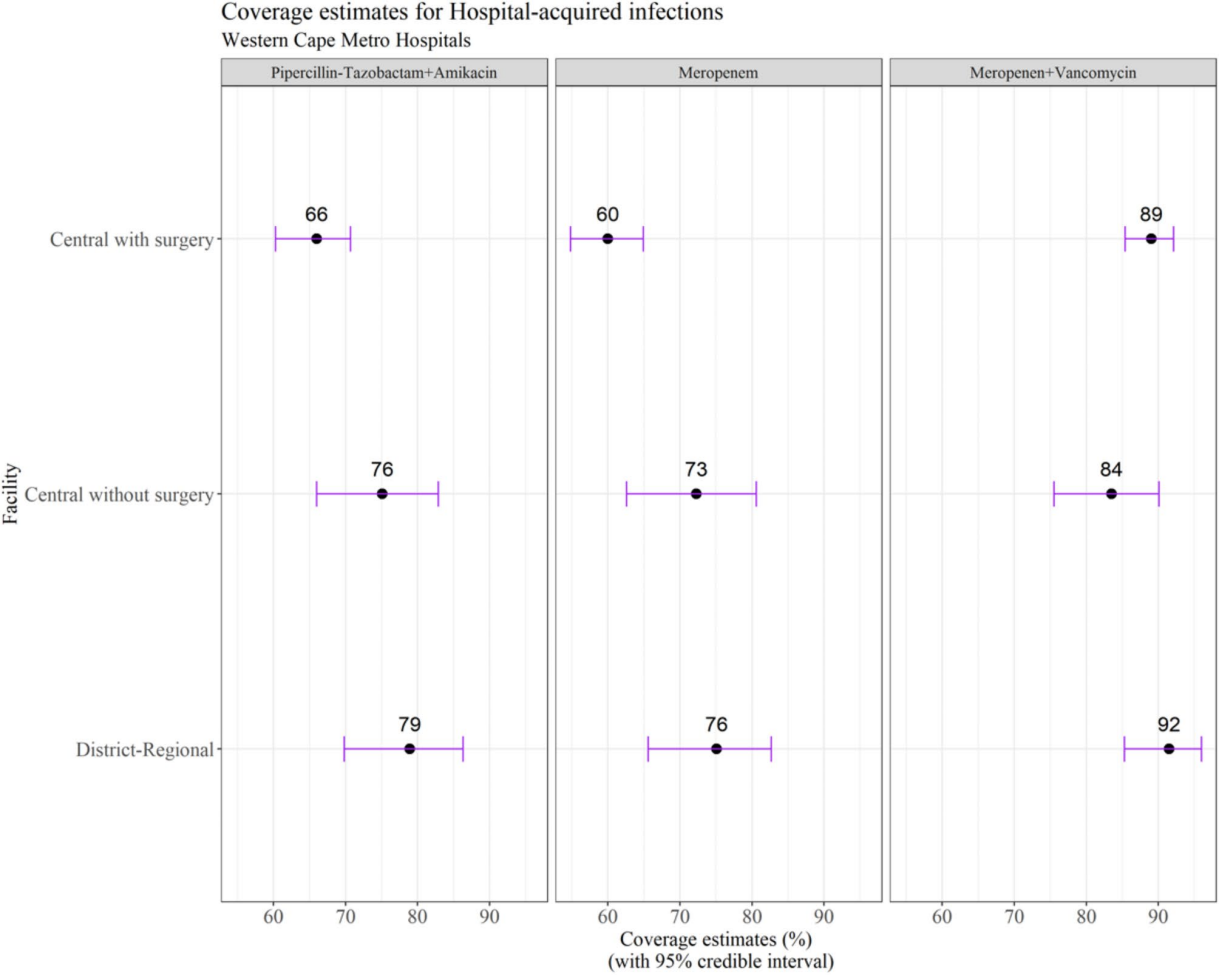
Coverage estimates for empiric antibiotic regimens for healthcare-associated bloodstream infection (HA-BSI) in Western Cape neonatal units (2017–2018)

For the WISCA analysis, the top 9 bacterial species were included (Table 2), representing 90.3% of the total isolates (496/549). Mean estimated empiric antibiotic coverage rates varied by unit type (central vs. district-regional): 66–79% for piperacillin-tazobactam plus amikacin, 60–76% for meropenem and 84–92% for meropenem plus vancomycin (Fig. 2).

## Discussion

HAI episodes are the most frequent healthcare complication encountered in LMIC neonatal units. In this 2-year review of HA-BSI epidemiology at nine South African neonatal units, we determined an overall HA-BSI rate of 2.0/1000 patient days, with a three-fold higher infection rate at the largest central neonatal unit with onsite surgery compared to the eight central, regional and district hospital neonatal units without onsite neonatal surgical care. Most HA-BSI episodes occurred between day 3–14 of life, with over two-thirds affecting extremely or very preterm infants. One-third of infants who acquired HA-BSI died, with 67% of deaths occurring within three days of infection onset. Gram-negative pathogens were responsible for almost two-thirds of HA-BSI episodes, and increased risk of death by 50% compared to Gram-positive HA-BSI episodes. Empiric antibiotic coverage rates for HA-BSI were sub-optimal at all neonatal units, with < 80% coverage for the most frequently used antibiotic regimens (piperacillin-tazobactam plus amikacin, and meropenem) against commonly encountered neonatal pathogens. Antibiotic discordance (bug-drug mismatch) was largely driven by carbapenem-resistant *A. baumannii* and methicillin-resistant *S. aureus* BSI.

The central hospital with onsite surgery had a three-fold higher HA-BSI rate compared to central, regional and district hospitals without onsite neonatal surgical care. Possible contributing factors to the higher infection rate at this unit may include higher rates of blood culture sampling with lower thresholds to submit laboratory investigations for sepsis screening, higher patient admission volumes, higher bed occupancy rates, longer hospital stays, and different disease profiles including complex surgical cases with a combined neonatal medical/surgical intensive care unit. Additional factors which could not be assessed were differences in infection prevention and control and antibiotic stewardship practices in obstetric and neonatal care between hospitals. Notwithstanding the variable HA-BSI rates documented at these neonatal units, the overall rate is markedly lower than that reported from other South African [15, 16] and African neonatal units [25–27].

The crude neonatal HA-BSI mortality rate of 32% observed far exceeds that previously reported from large systematic reviews (11–19%) [10, 11] and that reported from the large central neonatal unit (with on-site surgery) at 15.6% (between 2009 and 2013) [13]. Possible reasons for the higher HA-BSI mortality rates in recent years include worsening AMR rates [16, 28] leading to discordant antibiotic therapy, and increasing numbers of high-risk deliveries, with rising bed occupancy and neonatal unit overcrowding. Discordant antibiotic therapy (with mismatch between the pathogen’s antibiotic susceptibility and the empiric HA-BSI treatment regimen) has been shown to increase BSI-associated mortality rates three-fold [29]. The importance of HAI caused by AMR pathogens in the causal pathway in up to 70% of in-hospital preterm neonatal deaths has been highlighted in a post-mortem study at a large South African neonatal unit [12]. Improving infection prevention and control in neonatal care, especially for preterm neonates who have prolonged hospital stay, high rates of invasive device use and impaired immunity, skin and gut barriers, is critical as African countries target attainment of the SDG 3 goal to reduce the neonatal death rate below 12/1000 live births [30].

The major pathogens in this study, *K. pneumoniae*, *S. aureus* and *E. coli* are also important neonatal pathogens in other LMIC [27, 28] with Gram-negative pathogens predominating as causes of HA-BSI [31]. These three pathogens alone accounted for more than half of all neonatal HA-BSI episodes in this cohort, although the proportional contribution varied by neonatal unit type, with relatively higher rates of *S. aureus* BSI at the surgical-central unit and higher rates of *E. coli* BSI at the non-surgical central, regional and district units. *S. marcescens* and *A. baumannii* were other important pathogens at the three central neonatal units. Candida species (57% non-albicans) were infrequent HA-BSI pathogens overall (< 3%) in contrast to the more recently published BabyGERMS dataset where fungi accounted for 7% of BSI episodes in infants < 28 days of life nationally [16].

AMR is a growing concern in Gram-negative neonatal pathogens and existing World Health Organisation empiric antibiotic recommendations (ampicillin plus gentamicin or third generation cephalosporins) no longer provide adequate HA-BSI pathogen coverage in many LMIC neonatal units [32]. Despite this bug-drug mismatch, recent point prevalence surveys show that less than a quarter of neonates/children with sepsis globally are still prescribed the WHO-recommended first- or second-line empirical antibiotics for sepsis [33]. In this cohort, we produced antibiotic coverage estimates for three commonly used antibiotic regimens against the circulating neonatal HA-BSI pathogens. We demonstrated that the most frequently used antibiotic regimens (piperacillin-tazobactam plus amikacin, and meropenem) provided sub-optimal empiric coverage for HA-BSI at all neonatal units, with < 80% coverage of frequently encountered neonatal pathogens. Inclusion of vancomycin with meropenem empirically improved regimen coverage at all units above 80%, mostly owing to improvement in coverage of methicillin-resistant *S. aureus* and *E. faecium* HA-BSI. Given that these estimates are from a time preceding emergence of carbapenem resistance among Enterobacterales in the province’s neonatal units [34], it is likely that the estimated antibiotic coverage rates would be even lower now. Although this study focussed on the potential mortality impact of inappropriately narrow empiric antibiotic coverage, caution should be exercised in application of the findings to selection of empiric antibiotic therapy, to prevent overuse of broad-spectrum antibiotic therapy when not needed. For example, although addition of vancomycin improved empiric coverage rates in this cohort, vancomycin would be appropriate only in neonates with suspected HAI and risk factors for MRSA sepsis such as indwelling central lines, thrombophlebitis or skin and soft tissue infection.

Given the variability in pathogen, AMR and patient population by neonatal unit and region, it is critical that the neonatal HA-BSI AMR surveillance and antibiotic coverage estimates should be implemented to provide timely evidence to inform locally appropriate HA-BSI treatment recommendations. Repeated WISCA analysis will be valuable in estimating future antibiotic coverage of new and repurposed antibiotic combination regimens such as flomoxef, fosfomycin, colistin, and cefiderocol, among others.

This study has several important strengths including a modest degree of generalizability as a diverse multi-site study conducted at nine Western Cape neonatal units, including central, regional and district hospitals with a focus on a clearly defined form of infection (HA-BSI). Limitations of this study include the use of retrospective data which precluded collection of data on antibiotic treatment, site or source of infection, recent neonatal surgery, potential confounders, and variability in patient populations between neonatal units.

## Conclusion

Most HA-BSI events affected preterm and low birth weight neonates. HA-BSI burden was substantially higher at the central hospital with onsite surgery compared to others. One in three patients with HA-BSI died, with the highest risk of death in very preterm neonates and those with Gram-negative/fungal HA-BSI. Pathogen coverage of current empiric antibiotics is moderate and requires annual review given increasing carbapenem resistance levels in South African neonatal units.

## Electronic supplementary material

Below is the link to the electronic supplementary material.

Supplementary Material 1

## Data Availability

The datasets generated during and/or analysed during the current study are available from the corresponding author on reasonable request.

## Abbreviations

AMR: Antimicrobial resistant
CLSI: Clinical Laboratory Standards Institute
HA-BSI: Healthcare-associated bloodstream infections
IPC: Infection prevention and control
LMIC: Low- and middle-income countries
NHLS: National Health Laboratory Service
MERO: Meropenem
MERO+VANC: Meropenem plus vancomycin
PIP+AMIK: Piperacillin-tazobactam plus amikacin
US CDC: United States Centers for Disease Control
WISCA: Weighted-incidence syndromic combination antibiogram
