# Supplementary material for: Epidemiology of healthcare-associated bloodstream infection in South African neonatal units
Source: BMC Infect Dis. 2024 Nov 26;24:1350. doi: 10.1186/s12879-024-10219-0 (PMC11600642; doi:10.1186/s12879-024-10219-0)
Supplement: Supplementary file 1 — Supplementary Material 1 [file 12879_2024_10219_MOESM1_ESM.docx]

**Supplementary table: Susceptibility of neonatal healthcare-associated bloodstream infection (HA-BSI) pathogens to empiric antibiotic regimens***

*Table shows isolates covered by regimen (%) as total isolates susceptible /total isolates tested for each species, n (%)

PIP+AMIK = piperacillin-tazobactam plus amikacin

MERO = meropenem

MERO+VANC = meropenem plus vancomycin

|  | **Total neonatal units**  9 hospitals | | | **Medical and surgical unit**  1 hospital | | | **Medical units (offsite surgery)**  8 hospitals | | |
| --- | --- | --- | --- | --- | --- | --- | --- | --- | --- |
|  | **PIP+**  **AMIK** | **MERO** | **MERO+**  **VANC** | **PIP+**  **AMIK** | **MERO** | **MERO+**  **VANC** | **PIP+**  **AMIK** | **MERO** | **MERO+**  **VANC** |
| *K. pneumoniae* | 134/140 (95.7%) | 137/140 (97.9%) | 137/140 (97.9%) | 81/85 (95.3%) | 82/85 (96.5%) | 82/85  (95.3%) | 53/55 (96.4%) | 55/55 (100%) | 55/55  (100%) |
| *S. aureus* | 27/87 (31.0%) | 27/87 (31.0%) | 87/87  (100%) | 16/70 (22.9%) | 16/70 (22.9%) | 70/70  (100%) | 11/17 (64.7%) | 11/17 (64.7%) | 17/17  (100%) |
| *E. coli* | 55/56 (98.2%) | 56/56 (100%) | 56/56  (100%) | 26/26 (100%) | 26/26 (100%) | 26/26  (100%) | 29/30 (96.7%) | 30/30 (100%) | 30/30  (100%) |
| *S. marcescens* | 49/52 (94.2%) | 52/52 (100%) | 52/52  (100%) | 37/39 (94.9%) | 39/39 (100%) | 39/39  (100%) | 12/13 (92.3%) | 13/13 (100%) | 13/13  (100%) |
| *A. baumannii* | 11/42 (26.2%) | 9/42  (21.4%) | 9/42  (21.4%) | 8/31  (25.8%) | 7/31 (22.6%) | 7/31  (22.6%) | 3/11  (27.3%) | 3/11 (27.3%) | 3/11  (27.3%) |
| *E. faecalis* | 36/36 (100%) | 0/36  (0%) | 36/36  (100%) | 24/24 (100%) | 0/24  (0%) | 24/24  (100%) | 12/12 (100%) | 0/12  (0%) | 12/12  (100%) |
| *E. faecium* | 0/35  (0%) | 0/35  (0%) | 35/35  (100%) | 0/23  (0%) | 0/23  (0%) | 23/23  (100%) | 0/11  (0%) | 0/11  (0%) | 11/11  (100%) |
| *E. cloacae* | 22/26 (84.6%) | 25/26 (96.2%) | 25/26  (96.2%) | 17/20  (85%) | 19/20 (95%) | 19/20  (95%) | 5/6  (83.3%) | 5/6  (83.3%) | 5/6  (83.3%) |
| *Group B Streptococcus* | 22/22 (100%) | 22/22 (100%) | 22/22  (100%) | 10/10 (100%) | 10/10 (100%) | 10/10  (100%) | 12/12 (100%) | 12/12 (100%) | 12/12  (100%) |
| **Total fixed in vitro antibiotic coverage** | **357/496**  **(72.0%)** | **329/496**  **(66.3%)** | **458/496**  **(92.3%)** | **220/328**  **(67.1%)** | **199/328 (60.7%)** | **300/328 (91.5%)** | **137/167**  **(82.0%)** | **130/167 (77.8%)** | **158/167**  **(94.6.2%)** |
